# Intraoperative Blood Pressure and Carbon Dioxide Values during Aneurysmal Repair and the Outcomes after Aneurysmal Subarachnoid Hemorrhage

**DOI:** 10.3390/jcm12175488

**Published:** 2023-08-24

**Authors:** Umeshkumar Athiraman, Aaron J. Norris, Keshav Jayaraman, Abhijit V. Lele, Rainer Kentner, Preet Mohinder Singh, Omokhaye M. Higo, Gregory J. Zipfel, Rajat Dhar

**Affiliations:** 1 Department of Anesthesiology, Washington University, St. Louis, MO 63110, USA; 2 Department of Anesthesiology, University of Washington, Seattle, WA 98122, USA; 3 Departments of Neurological Surgery and Neurology, Washington University, St. Louis, MO 63110, USA; 4 Department of Neurology, Washington University, St. Louis, MO 63110, USA

**Keywords:** intraoperative blood pressure, end-tidal carbon dioxide, subarachnoid hemorrhage, cerebral vasospasm, delayed cerebral ischemia, neurologic outcomes

## Abstract

Cerebral autoregulation impairment is a critical aspect of subarachnoid hemorrhage (SAH)-induced secondary brain injury and is also shown to be an independent predictor of delayed cerebral ischemia (DCI) and poor neurologic outcomes. Interestingly, intraoperative hemodynamic and ventilatory parameters were shown to influence patient outcomes after SAH. The aim of the current study was to evaluate the association of intraoperative hypotension and hypocapnia with the occurrence of angiographic vasospasm, DCI, and neurologic outcomes at discharge. Intraoperative data were collected for 390 patients with aneurysmal SAH who underwent general anesthesia for aneurysm clipping or coiling between January 2010 and May 2018. We measured the mean intraoperative blood pressure and end-tidal carbon dioxide (ETCO_2_), as well as the area under the curve (AUC) for the burden of hypotension: SBP below 100 or MBP below 65 and hypocapnia (ETCO_2_ < 30), during the intraoperative period. The outcome measures were angiographic vasospasm, DCI, and the neurologic outcomes at discharge as measured by the modified Rankin scale score (an mRS of 0–2 is a good outcome, and 3–6 is a poor outcome). Univariate and logistic regression analyses were performed to evaluate whether blood pressure (BP) and ETCO_2_ variables were independently associated with outcome measures. Out of 390 patients, 132 (34%) developed moderate-to-severe vasospasm, 114 (29%) developed DCI, and 46% (169) had good neurologic outcomes at discharge. None of the measured intraoperative BP and ETCO_2_ variables were associated with angiographic vasospasm, DCI, or poor neurologic outcomes. Our study did not identify an independent association between the degree of intraoperative hypotension or hypocapnia in relation to angiographic vasospasm, DCI, or the neurologic outcomes at discharge in SAH patients.

## 1. Introduction

Aneurysmal subarachnoid hemorrhage (SAH) is a severe form of hemorrhagic stroke with high morbidity and mortality [1,2]. Outcome largely depends on two factors: (1) the initial bleeding severity and (2) secondary brain injury triggered by the bleeding. Delayed cerebral ischemia (DCI) is a form of secondary brain injury that plays a significant role in the patient’s outcome after SAH. DCI is characterized by several components, including (1) large-artery vasospasm; (2) microvessel thrombosis; (3) autoregulatory dysfunction; and (4) cortical spreading depolarization. Cerebral vasospasm and the DCI induced by cerebral vasospasm are the principal contributors for poor patient outcomes after SAH [2,3].

There are numerous studies demonstrating that blood pressure and end-tidal carbon dioxide (ETCO_2_) parameters play a crucial role in the patient’s outcome after SAH [4,5,6,7,8,9,10,11,12,13,14,15,16,17,18]. After SAH, intracranial pressure is acutely elevated, leading to global cerebral ischemia. Cerebral blood flow becomes blood pressure-dependent, and therefore, the maintenance of adequate blood pressure is critical to preserve cerebral perfusion [19]. Hypotension, both acutely and in the days after SAH, may be poorly tolerated, as it could lead to or worsen cerebral ischemia [4,5,6,7,8,9]. Earlier studies have linked intraoperative hypotension (measured via systolic blood pressure (SBP)) to an increased incidence of vasospasm and cerebral infarcts in SAH patients undergoing aneurysm clipping [6,7]. In contradistinction, two studies showed that intraoperative hypotension (measured through mean blood pressure (MBP)) was not associated with DCI or poor neurologic outcomes in SAH patients undergoing aneurysm repair [16,17].

Another major determinant of cerebral autoregulation and vasomotor tone is carbon dioxide, a potent and direct regulator of cerebral blood flow [18]. Previous studies have associated hypocapnia with vasospasm, DCI, and poor neurologic outcomes in mechanically ventilated SAH patients [11,12,13]. On the contrary, a recent observational study showed that intraoperative hypocapnia during aneurysm repair was not associated with DCI or poor neurologic outcomes in SAH patients [17]. The aim of our current study was to examine the impact of intraoperative blood pressure (using both systolic and mean blood pressures and calculating the burden of hypotension and not just the mean pressure) and ETCO_2_ variables on secondary brain injury and neurologic outcomes in SAH patients. Our hypothesis was that intraoperative hypotension and hypocapnia are associated with an increased incidence of angiographic vasospasm, DCI, and poor neurologic outcomes in SAH patients.

## 2. Materials and Methods

Institutional review board approval was obtained at Washington University in Saint Louis (Approval no—201610152) to conduct this retrospective study. Consecutive patients who presented for clipping/coiling of ruptured aneurysms after SAH from 1 January 2010 to 31 May 2018 were included in this study. A prospective neurocritical care/neurosurgical database and hospital charts were used to review the following details: patient demographics (age, gender, and family history), clinical presentation (Hunt–Hess grade, modified Fisher grade, aneurysm location and size), treatment modality and the outcome variables. The electronic anesthesia database was used to retrieve the following details: hemodynamic and ventilatory variables (intraoperative—average systolic blood pressure (SBP), diastolic blood pressure (DBP), mean blood pressure (MBP), and end-tidal carbon dioxide (ETCO_2_)) and calculate the area under the curve (AUC) for the following cutoffs—AUC SBP < 100 and >160 mmHg, AUC MAP < 65 and >100 mmHg, and AUC ETCO_2_ < 30 and >45 mmHg. The area under the curve was calculated by plotting the time–pressure graph. The plotted area was divided into the smallest possible rectangles or trapezoids. The summation of the area of these rectangles/trapezoids gave the area under the curve. The base of each tiny rectangle/trapezoid was formed by the standardized time axis (*X*-axis). The height of the trapezoids/rectangles was formed by the blood pressure value (*Y*-axis). The cumulative area was calculated for the above small quadrilaterals and represented the total area under the curve for the studied blood pressure, taking into account not only the value of the blood pressure but also the time for which it stayed. The majority of patients had arterial monitoring of blood pressure. ETCO_2_ was measured from induction to end-of-case using capnography. The few initial and final ETCO_2_ data points (values less than 10) were removed from the analysis for all the patients, as the ETCO_2_ values before intubation and after extubation may not reflect the actual ETCO_2_ values. Similarly, blood pressure outlier values were excluded from the analysis (values such as 0, and BP values above 200), as these likely represented artifacts as a result of leveling, or flushing the arterial line transducer. The intraoperative hemodynamic and ventilatory management of these patients were at the discretion of the attending anesthesiologist, and no specific study intervention was involved.

### 2.1. Outcome Measures

Angiographic vasospasm was classified into moderate (25–50% stenosis) to severe (>50% stenosis) narrowing of at least one major intracranial artery on a catheter angiogram [20]. The catheter angiogram was performed and interpreted by one of the three experienced interventional neuroradiologists. The most severely affected vessel was used for quantification of severity if vasospasm was noted in multiple vessels. Any degree of angiographic vasospasm and a decline in neurological status (either temporary or permanent) upon physician examination (including alertness, orientation, cranial nerve palsy, pronator drift, or focal motor deficit) or a decrease in Glasgow Coma Scale of ≥2 upon examination without other identifiable causes present (such as hydrocephalus, seizure, or fever) was defined as DCI. Good neurologic outcome was defined as a modified Rankin score (mRS) of (0–2) and poor outcome as an mRS of (3–6) at discharge.

### 2.2. Statistical Analysis

Statistical analysis was conducted with SPSS v. 23.0 (IBM Corp., Armonk, NY, USA). The Chi square test or Fisher exact test was used for univariate analysis for categorical variables, and the Mann–Whitney U test for continuous variables. Relevant blood pressure and ETCO_2_ values that appeared significantly associated with each outcome in the univariate analysis (*p* < 0.1) were entered into the multivariate model, adjusting for the known risk factors such as age, Hunt–Hess grade, modified Fisher grade, and type of treatment. A model parameter with *p* < 0.05 was considered statistically significant. A correlation matrix was constructed using Spearman’s rank correlation coefficient to assess the association between the interested BP/ETCO_2_ and outcome variables. The correlation values close to 1 indicate a strong association, 0 indicates no association, and −1 indicates a negative association between the assessed variables. Significant correlations are presented with the asterisk symbol (*). The number of asterisks increase with higher significance (lower *p* values).

## 3. Results

During the study period, we identified a total of 436 patients with the diagnosis of aneurysmal SAH. Out of these, 46 patients were excluded, as 24 patients did not receive a screening catheter angiography to assess for cerebral vasospasm for a variety of reasons, and data for the other 22 patients could not be retrieved. In our current study, a total of 390 patients (115 males and 275 females) with a mean age of 56 ± 15 years were included. Anterior circulation aneurysms accounted for 326 patients (84%), and posterior circulation aneurysms were found in 64 patients (16%). Surgical clipping was performed in 151 patients (39%), and endovascular coiling was performed in 239 patients (61%).

### 3.1. Angiographic Vasospasm

**Figure 1:**
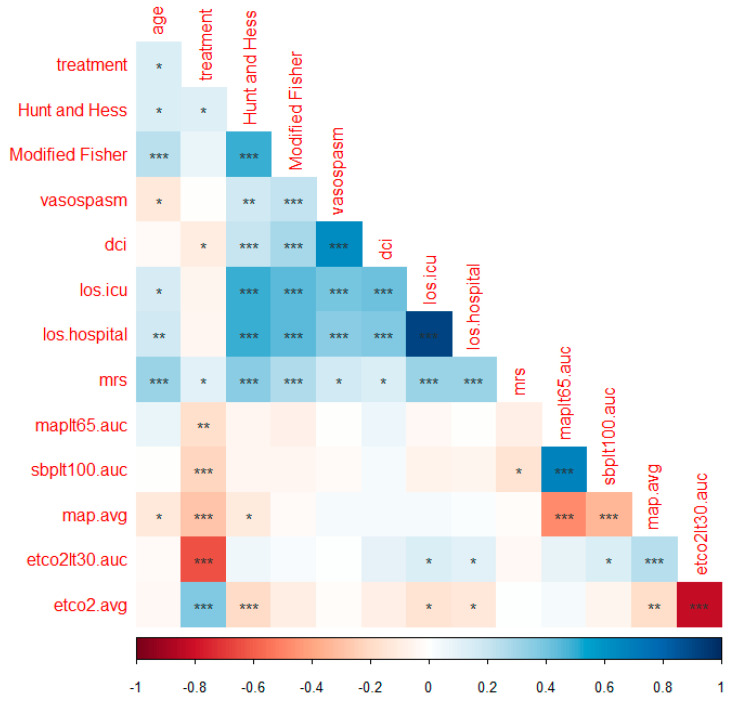
Correlation matrix. DCI = delayed cerebral ischemia. los = length of stay. mrs = modified Rankin scale. sbp = systolic blood pressure. map = mean arterial blood pressure. lt = less than. auc = area under curve. ETCO_2_ = end-tidal carbon dioxide. * *p* < 0.05, ** *p* < 0.01, *** *p* < 0.001.

Angiographic vasospasm occurred in 132 (34%) of the cohort and was associated with younger age, as well as higher Hunt and Hess and modified Fisher grades (Table 1). None of the intraoperative BP and ETCO_2_ variables were found to be significant in the univariate analysis. The interrelationships of intraoperative BP and ETCO_2_ variables with the measured outcomes are demonstrated in the correlation matrix (Figure 1).

### 3.2. Delayed Cerebral Ischemia (DCI)

DCI occurred in 114 (29%) of the cohort and was associated with higher modified Fisher and Hunt and Hess grades, as well as aneurysm treatment. Table 1 shows the univariate analysis comparing various characteristics in those with and without DCI. Though no intraoperative BP and ETCO_2_ variables were found to be significant in the univariate analysis, a trend in significance was noted for a burden of ETCO_2_ below <30 mm Hg and a lower average ETCO_2_. After adjusting for relevant clinical variables, both a burden of ETCO_2_ < 30 mm Hg (OR 0.983, CI 0.942–1.027, and *p* = 0.450) and the intraoperative average ETCO_2_ (OR 0.998, CI 0.926–1.075, and *p* = 0.952) were not associated with DCI.

### 3.3. Neurologic Outcome (mRS)

Of 370 patients with available mRS data at discharge, 169 (46%) had favorable outcomes. This was strongly associated with younger age and lower Hunt and Hess and modified Fisher grades. Table 1 shows the univariate analysis comparing various characteristics in SAH patients with good and poor clinical outcomes. A burden of SBP < 100 mm Hg and a lower intraoperative average SBP were associated with more favorable outcomes. However, neither physiologic measure was associated with outcome in multivariable analysis, largely due to the correlation of (and correction of) these with age.

## 4. Discussion

The key finding in our study is that all the measures of intraoperative hypotension and hypocapnia were not independently associated with the risk of angiographic vasospasm, DCI, or neurologic outcomes in SAH patients undergoing aneurysm repair by clipping or coiling. Despite earlier work suggesting such physiologic measures could influence delayed ischemia and outcomes, our findings are in accordance with two previously published retrospective studies showing that intraoperative blood pressure and ETCO_2_ values were not associated with DCI occurrence or poor neurologic outcomes at discharge and 3 months as measured by the GOS [16,17].

Our current study differs from the aforementioned studies in several ways: (1) We defined hypotension and hypertension with both SBP and MBP, whereas the other studies reported MBP only. Investigating the effects of SBP on SAH patients is important, as it is shown to be a critical variable representing the transluminal pressure across the aneurysm wall, and so it is a relevant target to prevent rebleeding [21]. In addition, it is important to note that a nationwide survey conducted among physicians and advanced practitioners who took care of SAH patients showed that the majority of practitioners prefer to monitor SBP to follow up on SAH patients [22]. (2) The impact of intraoperative hypercapnia has not been evaluated. This is essential, as controlled hypercapnia has been shown to improve cerebral blood flow in SAH patients, which could ameliorate ischemic deficits [14,15]. (3) We have an additional end point—cerebral vasospasm. And (4) we used a different measure to evaluate the neurologic outcome (mRS).

### 4.1. Blood Pressure and SAH

Blood pressure management after SAH is critical, and tight control is desired, as hypertension could result in rebleeding, and hypotension could result in DCI, leading to poor outcomes. Though previous guidelines by the American Heart Association and the neurocritical care society recommended an SBP management of less than 160–180 mmhg until the aneurysm is secured [19,23], current guidelines by the American Heart Association do not recommend any specific blood pressure target due to insufficient evidence [24]. A small retrospective study (with 84 patients) on SAH patients undergoing aneurysm clipping showed that intraoperative hypotension (defined as an SBP less than 90 mmHg for more than 15 min) was associated with vasospasm [6]. A follow-up study with a larger patient population (398 patients) identified intraoperative hypotension (described as a minimum 20% drop in SBP from the initial values or a 30 mmHg drop in SBP for more than 15 min) as an independent risk factor for the occurrence of postoperative cerebral infarction [7]. On the contrary, an observational study (with 164 patients) from Hoff et al. demonstrated that intraoperative hypotension (defined as the decrease in MAP of more than 30%, 40%, and 50% compared to the preoperative baseline blood pressure) was not associated with DCI or the poor outcomes as measured by the Glasgow outcome scale (GOS) in SAH patients undergoing aneurysm clipping [16]. Certainly, several differences exist between these studies. A patient selection bias (with more good-grade SAH patients in Hoff et al.’s study compared to the other two studies), the timing of surgery, outcomes measured, and the blood pressure component (SBP vs. MAP) utilized to determine the outcomes could all have possibly influenced the results.

Intriguingly, a retrospective study with a larger SAH cohort of 1099 patients evaluated the mean and time-weighted average area under the curve (AUC) for various absolute (MAP < 60, <70, <80, >90, and >100 mmHg) and relative thresholds (MAP < 70%, <60%, and <50%) and did not find an association between hypotension and neurologic outcomes at discharge and 3 months as measured by the GOS [17]. To note, this study included patients treated with both clipping and endovascular coiling, unlike the earlier studies. Our study measuring the AUC of blood pressure values (AUC SBP < 100 and >160 mmHg and AUC MAP < 65 and >100 mmHg) confirms this finding and also offers additional insight showing that hypo- or hypertension, quantified via both MAP and SBP, did not have a significant impact on angiographic vasospasm, DCI, or the neurologic outcomes in SAH patients undergoing aneurysm repair (clipping/coiling).

### 4.2. End-Tidal Carbon Dioxide and SAH

Carbon dioxide (CO_2_) is one of the major determinants of cerebral blood flow, and CO_2_ reactivity is generally preserved after SAH [18]. Currently, there are no guidelines for the management of PaCO_2_ or ETCO_2_ in SAH patients. A retrospective clinical study (with 102 patients) on mechanically ventilated SAH patients showed that the duration of hypocapnia (PaCO_2_ < 35 mmHg) was associated with symptomatic vasospasm and poor functional outcomes at 3 months as measured by the GOS [11]. A subsequent clinical study (with 207 patients) showed that spontaneous hyperventilation is common in SAH patients and associated with DCI and poor neurologic outcomes (mRS) at hospital discharge [13]. More recently, a retrospective study (with 244 patients) demonstrated that hypocapnia (PaCO_2_ < 30 mmHg) was associated with poor GOS outcomes at 3 months in SAH patients who were not critically ill and without concurrent intracerebral or intraventricular hemorrhage [12]. Alternatively, though hypercapnia is not directly indicated to improve outcomes in SAH, controlled hypercapnia with continuous CSF drainage/ICP monitoring was shown as a safe and feasible approach to enhance cerebral blood flow and brain tissue oxygen saturation in mechanically ventilated SAH patients [14,15]. To note, all the above-mentioned studies were carried out in an intensive care setting. Interestingly, a large retrospective study that evaluated the impact of blood pressure also examined the impact of intraoperative ETCO_2_ on SAH outcomes [17]. Akkermans et al. calculated the mean and time-weighted average area under the curve (AUC) for various absolute ETCO_2_ values (ETCO_2_ < 30, <35, <40, and <45 mmHg) and showed that intraoperative hypocapnia was not associated with poor neurologic outcomes in SAH patients undergoing aneurysm repair [17]. To note, the impact of hypercapnia was not evaluated in this study. Our study measuring the burden of hypo- and hypercapnia (AUC ETCO_2_ < 30 and >45 mmHg) validates these findings, showing that intraoperative hypocapnia does not have a significant impact on vasospasm, DCI, or the neurologic outcomes in SAH patients undergoing aneurysm repair. In addition, our study provides evidence that intraoperative hypercapnia does not have an impact on any of the measured SAH outcomes.

### 4.3. Limitations of the Study

Our study has several limitations: (1) It is a retrospective study design, which makes it impossible to disentangle causation from associations noted in observations with multiple potential confounders. (2) We reported ETCO_2_ and not PaCO_2_ values in our study. Studies have shown a good correlation between ETCO_2_ and PaCO_2_ values [25] and the presence of a smaller gradient (around 4 mmHg) in patients undergoing elective craniotomies [26]. So, we believe that this should not have a major influence on the outcomes. (3) It is possible that by selecting a few specific AUC cutoff points in our study, we may have missed an association between other blood pressure and ETCO_2_ values and SAH outcomes. And finally, (4) the impact of blood pressure and ETCO_2_ variables on long-term neurologic outcomes were not examined in the current study.

## 5. Conclusions

Overall, our study found that intraoperative hypotension and hypocapnia may not have a significant impact on angiographic vasospasm, DCI, or the neurologic outcomes in SAH patients. The observational nature of our study, showing an association but not causation, does not recommend a change in the current clinical management, until a more carefully designed prospective randomized trial sheds light on defining the optimal blood pressure and CO_2_ targets for the perioperative management of SAH patients.

## Figures and Tables

**Table 1:** Clinical, aneurysm, and hemodynamic characteristics by angiographic vasospasm, DCI, and mRS outcomes. Categorical variables are represented as numbers. Continuous variables are presented as median/IQR. *p* < 0.05 is statistically significant.

Characteristic	Angiographic Vasospasm (No) (n = 258)	Angiographic Vasospasm (Yes) (n = 132)	*p* Value	DCI (No) (n = 276)	DCI (Yes) (n = 114)	*p* Value	mRS Good Outcome (n = 169)	mRS Poor Outcome (n = 201)	*p* Value
Age (Median/IQR)	56 (48, 68)	52 (46, 61)	0.014	55 (47, 67)	54 (48, 64)	0.653	50 (44, 59)	60 (50, 71)	<0.0001
Male Gender n (%)	79 (31%)	36 (27%)	0.478	79 (29%)	36 (32%)	0.575	43 (25%)	63 (31%)	0.211
Family History n (%)	20 (8%)	9 (7%)	0.731	22 (8%)	7 (6%)	0.51	18 (11%)	11 (6%)	0.055
Anterior Circulation n (%)	214 (83%)	112 (85%)	0.631	229 (83%)	97 (85%)	0.608	139 (82%)	170 (85%)	0.623
Clipping n (%)	99 (39%)	52 (39%)	0.867	98 (36%)	53 (46%)	0.046	70 (41%)	72 (36%)	0.270
H and H Grading (Median/IQR)	3 (2, 3)	3 (2, 4)	0.001	3 (2, 3)	3/2 (2, 4)	<0.0001	2 (2, 3)	3 (2, 4)	<0.0001
Modified Fisher (Median/IQR)	3 (1, 4)	3 (3, 4)	<0.0001	3 (1, 4)	3/1 (3, 4)	<0.0001	2 (1, 3)	3 (2, 4)	<0.0001
Size of Ruptured Aneurysm (Median/IQR)	6 (4, 8)	5 (4, 8)	0.629	5 (4, 8)	6 (4, 8)	0.981	5 (4, 7)	6 (4, 8)	0.322
AUC SBP < 100 (mm Hg mins) (Median/IQR)	175 (16, 436)	138 (12, 424)	0.579	146 (14, 429)	166 (19, 451)	0.468	224 (24, 445)	97 (12, 419)	0.035
AUC SBP > 160 (mm Hg mins) (Median/IQR)	48 (0, 219)	42 (0, 200)	0.655	41 (0, 195)	54 (0, 275)	0.25	43 (0, 198)	49 (0, 229)	0.587
AUC MAP < 65 (mm Hg mins) (Median/IQR)	38 (0, 159)	36 (0, 114)	0.935	31 (0, 127)	50 (2, 176)	0.227	33 (2, 167)	33 (0, 124)	0.392
AUC MAP > 100 (mm Hg mins) (Median/IQR)	118 (14, 368)	128 (16, 474)	0.639	111 (10, 370)	145 (19, 488)	0.262	137 (34, 427)	117 (5, 408)	0.185
AUC ETCO_2_ < 30 (mm Hg mins) (Median/IQR)	133 (16, 620)	110 (19, 731)	0.954	118 (16, 533)	216 (21, 901)	0.068	92 (16, 757)	130 (20, 578)	0.689
AUC ETCO_2_ > 45 (mm Hg mins) (Median/IQR)	0 (0, 1)	0 (0, 1)	0.674	0 (0, 1)	0 (0, 2)	0.454	0 (0, 3)	0 (0, 0)	0.269
Intraoperative Average SBP (Median/IQR)	120 (112, 129)	120 (112, 127)	0.669	120 (111, 129)	119 (114, 127)	0.81	119 (109, 128)	121 (113, 130)	0.051
Intraoperative Average DBP (Median/IQR)	64 (57, 70)	64 (59, 70)	0.387	64 (57, 71)	63 (58, 68)	0.772	64 (59, 71)	63 (57, 70)	0.128
Intraoperative Average MBP (Median/IQR)	81 (75, 87)	81 (76, 88)	0.554	81 (75, 88)	82 (77, 85)	0.537	82 (76, 87)	80 (76, 87)	0.86
Intraoperative Average ETCO_2_ (Median/IQR)	31 (28, 33)	31 (29, 33)	0.819	31 (29, 33)	31 (28, 33)	0.09	31 (28, 33)	31 (29, 33)	0.933
COPD n (%)	24 (9%)	8 (6%)	0.270	26 (9%)	6 (5%)	0.174	11 (7%)	20 (10%)	0.240
HTN n (%)	161 (62%)	64 (49%)	0.008	167 (61%)	58 (51%)	0.08	85 (50%)	130 (65%)	0.006
Diabetes n (%)	35 (14%)	12 (9%)	0.199	36 (13%)	11 (10%)	0.349	15 (9%)	29 (14%)	0.104
CAD n (%)	24 (9%)	5 (4%)	0.05	22 (8%)	7 (6%)	0.531	13 (8%)	14 (7%)	0.779
Liver Failure n (%)	2 (1%)	1 (1%)	1.000	3 (1%)	0 (0%)	0.353	0 (0%)	3 (1%)	0.112

H and H = Hunt and Hess grading. AUC = area under curve. SBP = systolic blood pressure. DBP = diastolic blood pressure. MBP = mean blood pressure. COPD = chronic obstructive pulmonary disorder. HTN = hypertension. CAD = coronary artery disease.

## Data Availability

The data collected are unavailable to be shared publicly.
